# Supplementary material for: Scientific sinkhole: The pernicious price of formatting
Source: PLoS One. 2019 Sep 26;14(9):e0223116. doi: 10.1371/journal.pone.0223116 (PMC6763211; doi:10.1371/journal.pone.0223116)
Supplement: S1 Appendix — (DOCX) [file pone.0223116.s001.docx]

**S1 Appendix. Survey questions.**

The following questions relate to your occupation. We require your estimated income to calculate the hourly cost attributable to formatting. Although we understand this is a personal question, your answer will be kept anonymous and used only to calculate costs related to formatting.

1. What best describes your occupation in 2017 [UNIQUE ANSWER]
   1. Clinician/health care provider (e.g., medical doctor, nurse)
   2. Scientist/researcher (e.g., professor, scientist, post-doctoral fellow)
   3. Research assistant/research manager
   4. Student (e.g., undergraduate, masters, doctoral)
   5. I don’t work in science/academia [END SURVEY]
2. What was your gross personal income in 2017 (please estimate in U.S. dollars; if unsure, please enter the currency in which you are paid). You can access a currency converter here: <https://www.xe.com/currencyconverter/>
   1. >$20,000 per year
   2. $21,000-40,000 per year
   3. $41,000-60,000 per year
   4. $61,000-80,000 per year
   5. $81,000-100,000 per year
   6. $101,000-120,000 per year
   7. $121,000-140,000 per year
   8. $141,000-160,000 per year
   9. $161,000-180,000 per year
   10. $181,000-200,000 per year
   11. >$201,000 per year
   12. ____ Other (specify currency)

The following questions refer to manuscripts that were **published in 2017**. When we ask about formatting, we are referring to all time related to formatting the body of the manuscript, figures, tables, supplementary files, and references. DO NOT count time spent on statistical analysis, writing, or editing.

1. How many manuscripts were you responsible for submitting and/or formatting for publication in a peer-reviewed journal?
   1. _____
   2. 0 [END SURVEY]
2. On average, how long did you spend formatting your manuscript for submission (e.g., formatting references, tables, figures, headings etc.)?
   1. _____
      1. hours
      2. minutes
3. On average, how long did you spend formatting each paper from the time it was accepted for publication, until the time it was published? Do not count time spent re-formatting for submission to another journal.
   1. _____
      1. hours
      2. minutes
4. On average, how many journals did you have to submit to before having your manuscript accepted for publication?
   1. _____
5. On average, how long did you spend re-formatting your manuscript for re-submission (i.e., for a new journal after the paper was rejected)? Include all the time you spent re-formatting until the paper was accepted for publication, or you decided that your results were unpublishable and you terminated the project.
   1. _____
      1. hours
      2. minutes

Finally, some questions on general demographic information.

1. What is your age?
   1. _____ years
2. What is your gender?
   1. Woman
   2. Man
   3. Other
3. What country do you work in the majority of the time?
   1. [textbox]
