# Supplementary material for: Scientific sinkhole: The pernicious price of formatting
Source: PLoS One. 2019 Sep 26;14(9):e0223116. doi: 10.1371/journal.pone.0223116 (PMC6763211; doi:10.1371/journal.pone.0223116)
Supplement: S1 Table — (DOCX) [file pone.0223116.s002.docx]

**S1 Table. Outcomes related to cost of formatting for scientific publications, by age group.**

| Outcome (median, MAD) | Per manuscript | Per person, per year |
| --- | --- | --- |
|  | ≤37 years >37 years | ≤37 years >37 years |
| Number of manuscripts responsible for submitting and/or formatting per year | 3 (3.0) 4 (3.0)* | - |
| Number of submissions before publication | 2 (1.5) 2 (0) | - |
| Hours |  |  |
| Time spent on initial formatting | 4 (3.0) 4 (3.0) | 12 (10.4) 20 (18.9)* |
| Time spent re-formatting for re-submission | 3 (3.0) 3 (3.0) | 6 (5.9) 6 (5.9) |
| Total time spent formatting from initial submission until publication | 13.8 (10.7) 15.5 (11.1) | 40.5 (37.8) 69.5 (65.2)* |
| Cost |  |  |
| Wage-cost (US$) | $292 $717* | $938 $3669* |

*p<0.05.

A median split was used to create both age groups.
