# Supplementary material for: Scientific sinkhole: The pernicious price of formatting
Source: PLoS One. 2019 Sep 26;14(9):e0223116. doi: 10.1371/journal.pone.0223116 (PMC6763211; doi:10.1371/journal.pone.0223116)
Supplement: S2 Table — (DOCX) [file pone.0223116.s003.docx]

**S2 Table. Outcomes related to cost of formatting for scientific publications, by gender.**

| Outcome (median, MAD) | Per manuscript | Per person, per year |
| --- | --- | --- |
|  | Men Women | Men Women |
| Number of manuscripts responsible for submitting and/or formatting per year | 4 (3.0) 4 (3.0) | - |
| Number of submissions before publication | 2 (1.5) 2 (0) | - |
| Hours |  |  |
| Time spent on initial formatting | 4 (3.0) 5 (4.4) | 16 (14.8) 15 (13.3) |
| Time spent re-formatting for re-submission | 3 (2.2) 3 (3.0) | 8 (7.4) 6 (5.9) |
| Total time spent formatting from initial submission until publication | 13 (10.4) 14 (10.4)* | 60 (59.3) 50 (44.5) |
| Cost |  |  |
| Wage-cost (US$) | $467 $493 | $2015 $1726* |

*p<0.05.
