# Supplementary material for: Scientific sinkhole: The pernicious price of formatting
Source: PLoS One. 2019 Sep 26;14(9):e0223116. doi: 10.1371/journal.pone.0223116 (PMC6763211; doi:10.1371/journal.pone.0223116)
Supplement: S3 Table — (DOCX) [file pone.0223116.s004.docx]

**S3 Table. Outcomes related to cost of formatting for scientific publications, by occupation.**

| Outcome (median, MAD) | Per manuscript | Per person, per year |
| --- | --- | --- |
|  | Scientist Other | Scientist Other |
| Number of manuscripts responsible for submitting and/or formatting per year | 4 (3.0) 3 (1.5)* | - |
| Number of submissions before publication | 2 (1.5) 2 (1.5) | - |
| Hours |  |  |
| Time spent on initial formatting | 4 (3.0) 5 (4.4)* | 16 (14.8) 12 (11.9)* |
| Time spent re-formatting for re-submission | 3 (2.2) 3 (3.0) | 6 (5.9) 6 (6.7) |
| Total time spent formatting from initial submission until publication | 14 (10.4) 14 (11.9) | 56 (53.4) 48 (47.4)* |
| Cost |  |  |
| Wage-cost (US$) | $546 $314 | $2391 $947* |

*p<0.05.
